# Dibenzo[1,2,5]thiadiazepines Are Non-Competitive GABA_A_ Receptor Antagonists

**DOI:** 10.3390/molecules18010894

**Published:** 2013-01-11

**Authors:** Juan F. Ramírez-Martínez, Rodolfo González-Chávez, Raquel Guerrero-Alba, Paul E. Reyes-Gutiérrez, Roberto Martínez, Marcela Miranda-Morales, Rosa Espinosa-Luna, Marco M. González-Chávez, Carlos Barajas-López

**Affiliations:** 1 División de Biología Molecular, Instituto Potosino de Investigación Científica y Tecnológica, San Luis Potosí 78216, Mexico; E-Mails: francisco.martinez@uaslp.mx (J.F.R.-M.); biogigio@hotmail.com (R.G.-A.); respinosa@ipicyt.edu.mx (R.E.-L.); 2 Facultad de Ciencias Químicas, Universidad Autónoma de San Luis Potosí, San Luis Potosí 78210, Mexico; E-Mails: rodolfo.gonzalez@uaslp.mx (R.G.-C.); pa_edu3@yahoo.com.mx (P.E.R.-G.); 3 Instituto de Química, Universidad Nacional Autónoma de México, Coyoacán 04510, Mexico; E-Mail: robmar@servidor.unam.mx; 4 Departamento de Neurobiología Celular y Molecular, Instituto de Neurobiología, Universidad Nacional Autónoma de México, Querétaro 76230, Mexico; E-Mail: mmirandam@unam.mx

**Keywords:** dibenzothiadiazepines, GABA_A_ receptor antagonists, patch clamp, neurochemistry, biological activity, enteric neurons, electrophysiology

## Abstract

A new process for obtaining dibenzo[c,f][1,2,5]thiadiazepines (DBTDs) and their effects on GABA_A_ receptors of guinea pig myenteric neurons are described. Synthesis of DBTD derivatives began with two commercial aromatic compounds. An azide group was obtained after two sequential reactions, and the central ring was closed via a nitrene to obtain the tricyclic sulfonamides (DBTDs). Whole-cell recordings showed that DBTDs application did not affect the holding current but inhibited the currents induced by GABA (I_GABA_), which are mediated by GABA_A_ receptors. These DBTDs effects reached their maximum 3 min after application and were: (i) reversible, (ii) concentration-dependent (with a rank order of potency of **2c** = **2d** > **2b**), (iii) mediated by a non-competitive antagonism, and (iv) only observed when applied extracellularly. Picrotoxin (which binds in the channel mouth) and DBTDs effects were not modified when both substances were simultaneous applied. Our results indicate that DBTD acted on the extracellular domain of GABA_A_ channels but independent of the picrotoxin, benzodiazepine, and GABA binding sites. DBTDs used here could be the initial model for synthesizing new GABA_A_ receptor inhibitors with a potential to be used as antidotes for positive modulators of these receptors or to induce experimental epilepsy.

## 1. Introduction

**Figure 1:**
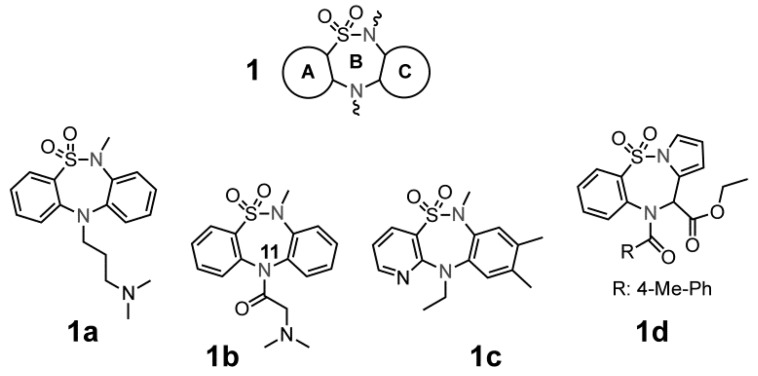
General chemical structure of the dibenzo[*c,f*][1,2,5]thiadiazepines **1**, and several DBTDs reported to have biological activity (compounds **1a**, **1b**, **1c**, and **1d**).

The synthesis of tricyclic compounds with a central thiadiazepine ring (see Figure 1, **1** ring B) were first described by Weber [1], followed by a description of the compounds anti-depressive effects (compound **1a**) [2,3]. In 1991, Giannotti *et al.* [4] prepared DBTD (compound **1b**) structural variants at nitrogen 11 (N-11) with the purpose of increasing the antidepressive effects previously observed by Weber, while reducing possible side effects. In addition to the effects of DBTDs on the central nervous system, these substances were found to act as non-nucleosidic reverse transcriptase inhibitors of HIV-1 (compound **1c**) [5] and to have anti-proliferative activity on leukemia cell lines (compound **1d**) [6], effects that increased the attention toward these tricyclic compounds.

**Figure 2:**
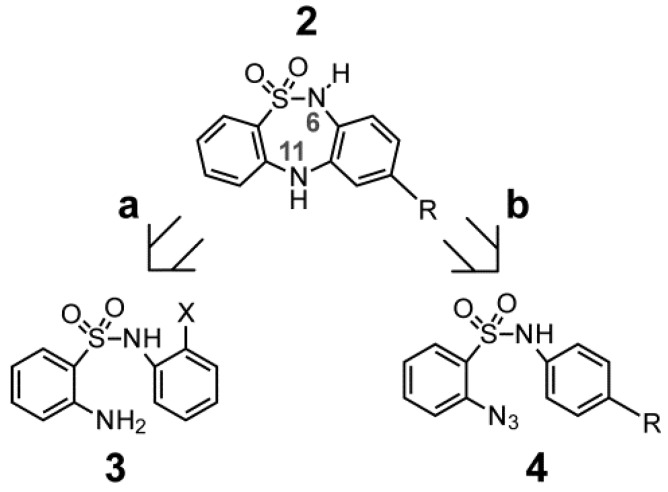
Retrosynthetic analysis of the non-substituted DBTDs. (**a**) Classical method for obtaining **2** by the Goldberg methodology; (**b**) Obtaining **2** from an aryl azide.

The central ring of DBTDs has been synthesized via the Goldberg method, which involves an Ullmann intra-molecular condensation reaction (N-C) (Figure 2, route a) [7]. This reaction is limited by the fact that *ortho*-haloanilines significantly reduce the number of possible substituents that may be included in the DBTDs. Only one report has described the use of this methodology for obtaining compounds **2a** and **2d** [8]. As an alternative approach, N-C bonds may be formed via intra-molecular reactions of aryl azides with benzene derivatives (Figure 2, route b), which has been described during the formation of carbazoles through thermolysis [9], photolysis [10], and recently, via metal catalysis [11,12]. The advantage of using aryl azides is that C-N bonds are formed directly. Therefore, the use of monosubstituted anilines with diverse functional groups can lead us toward obtaining DBTDs with functional variations in the C ring. Known DBTDs and triheterocyclic analogous compounds include diverse substituents on the nitrogen of the thiadiazepine ring (Figure 1); however, no studies have examined the biological activities of the parent compound and 9-substituted derivatives (Figure 2).

**Figure 3:**
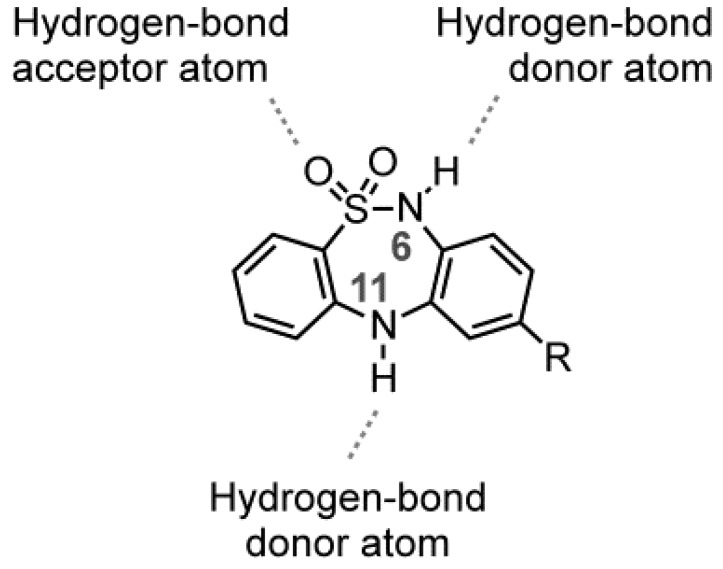
The dotted lines in DBTDs indicate probable interactions between hydrogen bonds and proteins.

This work is the first report to consider a biological study of DBTDs without substituents on nitrogens 6 and 11 of B ring, which were obtained through a process distinct from the Ullmann method. The presence of a hydrogen atom at N-6 and N-11 in a DBTD can determine the compound’s affinity toward proteins via hydrogen bond interactions [13], as shown in Figure 3.

**Scheme 1:**
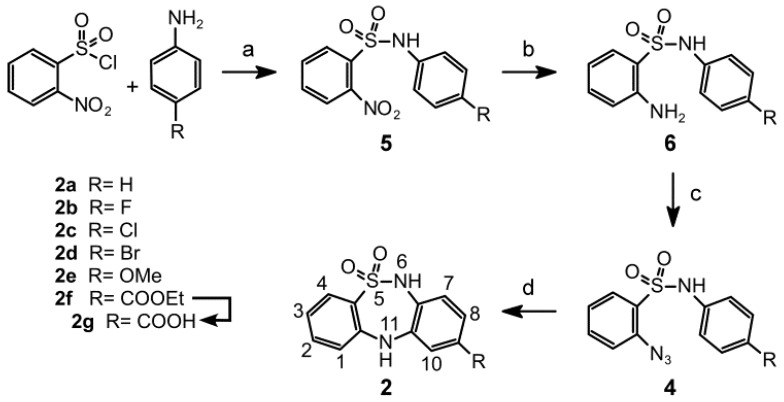
*Reagents and conditions*: (**a**) Anhydrous pyridine, dry acetone, N_2(g)_, 24 h; (**b**) SnCl_2_·2H_2_O, ethyl acetate, 4 h; (**c**) first step: NaNO_2_, F_3_CCO_2_H, 1 h; second step: NaN_3_, 1 h; (**d**) (C_6_H_5_)_2_O, 208 °C, 5 min. Compound **2g** was obtained from **2f**: first step: KOH 10%, 1 h.

The linear synthetic route to the DBTDs **2a**–**g** comprises four stages and proceeds as described in Scheme 1. The thiadiazepine ring is formed through direct amination of the C ring via intramolecular thermal cyclization of **4** (Figure 2, route b). This methodology provides an alternative to the classical amination of Goldberg approach, with respect to the formation of ring B in the substituted DBTDs (Figure 2, route a) [2,4,5,8,14]. DBTDs with a modified B ring were shown to have biological effects. Giannotti *et al.* synthesized a series of DBTDs with substituents in the thiadiazepine ring, and they showed that the compounds displayed a potential antidepressive effect using the apomorphine-induced hypothermia test [4]. However, these authors found no binding of DBTDs with receptors to dopamine, serotonin, histamine, benzodiazepine, GABA, acetylcholine, and adrenaline, and reported that DBTDs lack of effect on serotonin and noradrenaline uptake. However, such observations does not rule out that DBTDs might be modulating any of these receptor proteins through a different binding site than the one directly activated by a given agonist or modulator. At least three observations indicated us that GABA_A_ channels might be the target for DBTDs: (i) the tricyclic sulfonamide **2** is structurally similar to the 1,4-benzodiazepines, which are major positive modulators of these channels [15]; (ii) DBTDs have antidepressive actions [4] and (iii) GABA_A_ channels have been implicated in mood disorders, including depression [16,17]. Therefore, the aim of the present study was to further investigate the effects of DBTDs on GABA_A_ channels and to report a new synthetic platform for obtaining DBTD compounds that do not include the thiadiazepine ring substitutions. We found that these compounds inhibit directly GABA_A_ channels by a mechanism that is independent of the binding sites for GABA, picrotoxin, and benzodiazepine.

## 2. Results and Discussion

### 2.1. Chemistry

The synthetic route begins with the reaction of 4-substituted-anilines with 2-nitrobenzenesulfonyl chloride under reflux using pyridine as the base and acetone as solvent. The 2-nitrosulfonamides **5a**–**f** were obtained in good yields (66%–88%). The subsequent catalytic hydrogenation of the nitro group using tin(II) chloride under reflux with ethyl acetate yielded the amines **6a**–**f** in yields above 88%. The amino compounds were transformed to the corresponding 2-azidobenzensulfonamides through their diazotization with sodium nitrite in trifluoroacetic acid (note that the transformation of **6e** was accomplished using hydrochloric acid). The *in situ* substitution of the diazo group with sodium azide induced conversion to **4a**–**f** with a 78%–87% yield. In the final step, the thiadiazepine formation reaction proceeded via thermolysis above 208 °C in diphenyl ether, this temperature favoured the formation of an intermediate nitrene reagent [9]. A direct N-C-type amination of the C ring provided the DBTDs **2a**–**f** with yields of 67%–85%, except for compound **2f**, which was isolated in a yield of 12%. Compound **2g** was obtained by basic hydrolysis of **2f** after a hydrochloric acid treatment.

### 2.2. Biological Results

The inhibitory effects of DBTDs on the native GABA_A_ receptors of guinea pig myenteric neurons were studied here for the first time. The Cl^−^ concentrations outside and inside the neurons were similar, and a holding potential was −60 mV. At this potential, GABA (0.03–3 mM) induced inward currents (I_GABA_) in 86% of myenteric neurons. The amplitude of the currents was concentration-dependent (EC_50_ = 115 ± 10 μM) and varied among the different neurons with a range of 0.1–6 nA, in response to 300 μM GABA. Most neurons maintained a stable value of I_GABA_ during repeated GABA applications. Otherwise, the data were rejected. In order to test that these GABA currents are mediated by GABA_A_ channels bicuculline (0.1–100 μM) and picrotoxin (3–1,000 μM) were used, inhibitors of these receptors [18,19,20]. Both substances inhibited I_GABA_ in a concentration-dependent manner (data not shown) with an IC_50_ of 10 ± 2 and 6 ± 1 μM, respectively. Maximal concentrations used for both antagonists virtually abolished I_GABA_, as it was previously reported [18,19,20].

**Figure 4:**
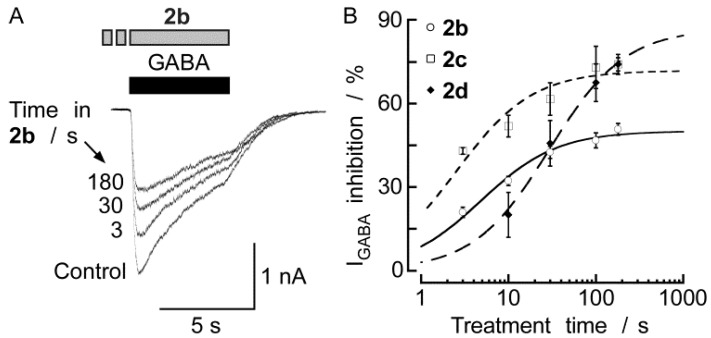
Halogenated DBTDs inhibited the I_GABA_ in a time-depended manner. (**A**) I_GABA_ was recorded before, during application of 100 μM **2b**, and after removal of the inhibitor of a given myenteric neuron for various lengths of time. The horizontal bars above the traces indicate the application profiles of the indicated substances. (**B**) Time course of I_GABA_ (induced by 300 μM GABA) inhibition induced by **2** over 3–180 s. Michaelis–Menten fits. Each data point represents the mean value from 3–12 different experiments. The lines represent the SEM.

Figure 4 shows that application of DBTDs (100 μM) inhibited the currents induced by GABA (300 μM) in a time-dependent manner (3–180 s) with time constants (*t*) of 4.9, 2.6, and 29.3 s for **2b**, **2c**, and **2d**, respectively. These constants were calculated by fitting the data using the Michaelis–Menten equation (*R^2^* = 0.98, 0.88, and 0.99, respectively). The maximum inhibition induced by **2b** and **2c** (100 μM) was reached 3 min after initial exposure. For **2d**, the time required to reach the maximum inhibition was calculated to be 17 min; however, the experimental maximum inhibition observed after a 3 min treatment was 74.2 ± 2.4% (n = 10), similar to the calculated maximum inhibition (87.0 ± 3.0%). In all subsequent experiments, a DBTD treatment time of 3 min was used. The current inhibition induced by **2b**, **2c**, and **2d** was fully reversed within five minutes of washing. The holding current remained constant in the presence of the compounds at all concentrations tested, indicating that the compounds could not open the GABA_A_ receptors or any other neuronal ion channel under the experimental conditions.

Control experiments were conducted using DMSO, the solvent used for all DBTDs, demonstrating that DMSO did not modify the properties of I_GABA_ alone at the maximum concentration used here, 0.33% V/V (data not shown). The inhibitory activities of the DBTDs were likely to be use-independent because their effects did not require active GABA_A_ receptors and the inhibitory activity increased over time, despite the absence of the agonist (GABA) [21]. This indicates that DBTDs bind to these channels during their closed stage.

**Figure 5:**
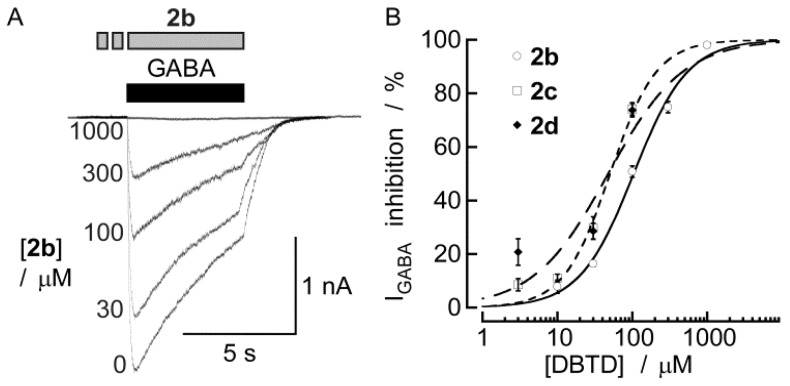
DBTDs inhibited I_GABA_ in a concentration-dependent manner. (**A**) Representative I_GABA_ recordings from a myenteric neuron in the presence of various concentrations of **2b**, which was added 3 min before GABA application. (**B**) Concentration—response curves for the effects of the DBTDs on the amplitude of I_GABA_. The lines indicate fits to the experimental data using a two-parameter logistic function, [22] assuming an inhibition of 100%. Each data point represents the mean ± SEM from 3–12 individual experiments.

The inhibitory effects of seven DBTDs at a 100 μM concentration are listed in Table 1. The data indicate that the rank order of potency for these inhibitors was **2c** = **2d** > **2b**; we were unable to calculate the potency of **2f**, **2e**, **2a**, **2g** due to solubility problems. Figure 5 shows the concentration–response curves for the inhibitory effects of three DBTDs (3–1,000 μM) on the currents induced by GABA (300 μM). The maximum effect of **2b** was achieved at 1 mM (inducing a complete inhibition of the GABA-activated currents), and an IC_50_ value (104 ± 9.2 μM). We did not reach the maximum inhibition for **2c** and **2d** because the compounds were insoluble at concentrations exceeding 300 μM under our experimental conditions. Curve fits were obtained in both cases with IC_50_ values of 50.4 ± 6.4 μM and 47.6 ± 30.3 μM for **2c** and **2d**, respectively.

**Table 1:** Percent inhibition in the presence of 100 μM compounds on GABA-induced inward currents, p IC_50_, cLog *P*, and physical data.

No.	Percentage of inhibition ^[a]^	p IC_50_/M	cLog *P* ^[b]^	Yield/%	mp/°C
2a	28.4 ± 1.4 (5)	ND	2.18	69	198
2b	50.8 ± 2.1 (12)	3.98	1.50	70	202
2c	74.2 ± 3.6 (6)	4.30	2.69	79	248
2d	74.2 ± 2.4 (10)	4.32	2.97	85	250
2e	43.7 ± 3.8 (3)	ND	1.92	67	171
2f	47.1 ± 3.7 (7)	ND	2.25	12	227
2g	16.0 ± 2.4 (6)	ND	1.87	100	350

[a] Values are given as the mean ± SEM, with the number of experiments in parentheses; [b] Data generated using HyperChem.

**Figure 6:**
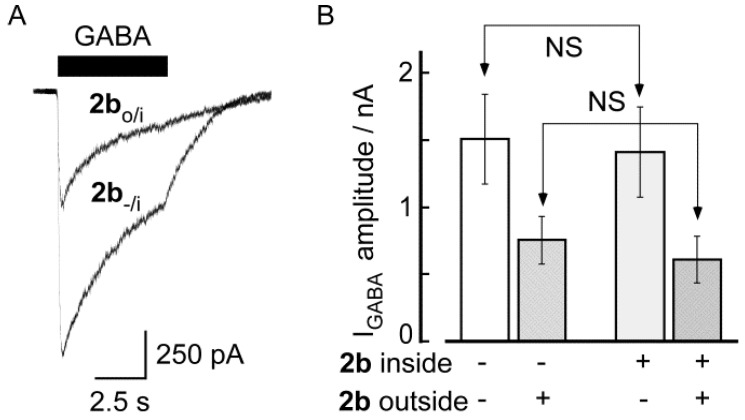
The inhibitory effects of **2b** on GABA_A_ receptors were mediated by an extracellular binding site. A) I_GABA_ for a 100 μM concentration of **2b** in the pipette (**2b**_-/i_), 10 min after obtaining the whole cell. I_GABA_ was recorded before (-/i) and in presence of extracellular (o/i) **2b** (100 μM for 3 min). B) Bars indicate the average amplitude of I_GABA_, and the lines above indicate the SEM. I_GABA_ amplitude or the inhibitory effect of **2b** did not differ significantly (NS) by the presence of **2b** inside the pipette. Statistical comparison of the data was done using the unpaired Student’s *t*-test.

We considered the possibility that because these novel substances were highly lipophilic (log *P* ~ 2.0 for all compounds, Table 1), the DBTDs could permeate the neuron membrane and interact with the inner part of the GABA_A_ channel, thereby inhibiting I_GABA_. To investigate this possibility, we added 100 μM **2b** to the pipette solution (internal) and monitored I_GABA_, and we applied **2b** to the outside of the cells and monitored the inhibitory effects (Figure 6). Experiments were performed using **2b**, even though cLog *P* was less than 2, because **2c** and **2d** were insoluble under the conditions employed. The amplitude of I_GABA_ (300 μM) for neurons with **2b** (100 μM) applied inside (−1408 ± 344 pA; n = 4) and measured 2 to 3 min after obtaining the whole cell configuration did not differ from the amplitude of the control I_GABA_ (−1510 ± 333 pA; n = 12) of experiments in which **2b** was tested extracellularly. Consistent with these findings, in recordings with **2b** in the pipette, the amplitude of I_GABA_ was the same 5 min (−1536 ± 379 pA) and 10 min (−1604 ± 409 pA; n = 4) after obtaining the whole-cell configuration. In addition, the presence of **2b** inside the neurons did not affect the magnitude of the inhibition induced by the extracellular application of **2b** (100 μM). Thus, such an inhibition was as large as that observed without **2b** inside the cells (Figure 6B). Altogether, these data rule out that DBTDs inhibitory effects on GABA_A_ channels are mediated by an intracellular target and therefore, they must be acting on the extracellular domain.

**Figure 7:**
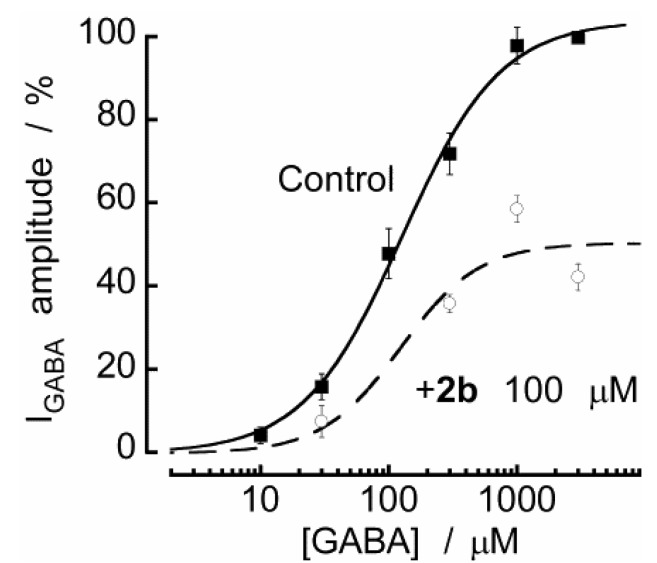
**2b** inhibits I_GABA_ in a non-competitive manner. (**A**) Concentration–response curves for GABA in the absence (Control) and in the presence of **2b**. Responses were normalized with respect to the curves obtained in the presence of 3 mM GABA in each cell and in the absence of **2b**. Each point represents the mean ± SEM for 5–12 neurons. The lines indicate fits of experimental data to a three-parameter logistic function.

Figure 7 shows two concentration-response curves for the effects of GABA, one in the absence and the other in the presence of 100 μM **2b**. As shown, the antagonistic effect of **2b** is not surmounted by increasing the GABA concentration. Indeed, the EC_50_ values for these curves were 127 ± 16 and 123 ± 84 μM in the absence and in the presence of **2b**, whereas, the maximum inhibition clearly decreased in the presence of **2b** (~50%) across the full GABA concentration–response curve. Our data demonstrate that the pharmacological antagonism by which **2b** inhibits the GABA_A_ receptors is non-competitive and therefore, it is unlikely acting at the GABA binding site. This is in agreement with a previous study [4] that shows no binding of DBTDs to GABA receptors.

**Figure 8:**
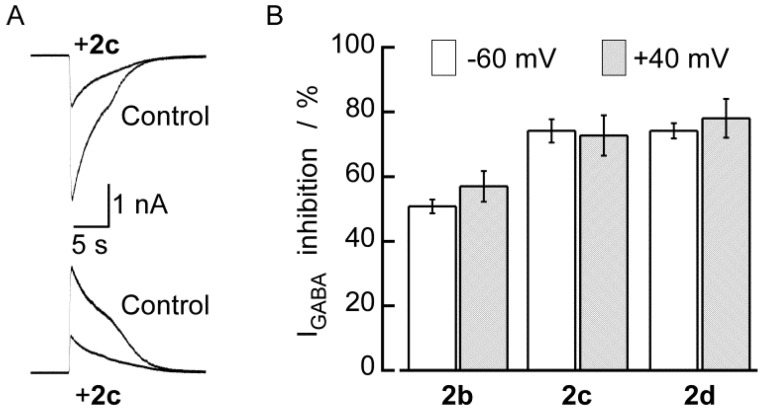
The inhibitory effects of compounds 2 on GABA_A_ channels were voltage independent. A) I_GABA_ induced by 300 μM GABA without (Control) or in presence of **2c** (100 μM) at −60 mV (upper traces) and +40 mV (lower traces) from the same neuron. I_GABA_ was recorded at 5 min intervals, and **2c** was applied 3 min before the second GABA application. B) The average (bars) inhibitory effect of **2b**, **2c**, and **2d** was the same at both membrane potentials. Lines over the bars indicate the SEM.

We further investigated if the inhibitory effect of DBTD on I_GABA_ was voltage dependent by conducting experiments at two holding membrane potentials, −60 mV and +40 mV in the same neurons. I_GABA_ (300 μM) was recorded in the absence or presence of 100 μM **2b**, **2c**, and **2d**. As shown in Figure 8, the inhibitory effects induced by any of the three substances were identical for an inward I_GABA_ (recorded at −60 mV) than for the outward I_GABA_ (recorded at +40 mV). These results indicate that the DBTDs inhibit I_GABA_ via a voltage-independent mechanism.

**Figure 9:**
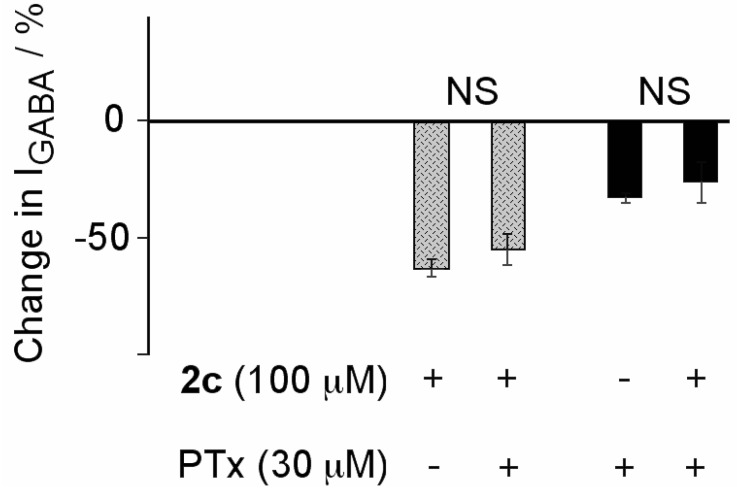
**2c**-induced inhibition of GABA_A_ channels was unaffected by picrotoxin (PTx). Bars and lines on their top, are means and SEM (n = 4). Statistical comparison of each pair of bars was done using the paired Student’s *t*-test. P values and non significant (NS) differences are indicated.

The fact that binding of compounds **2** on GABA_A_ channels can occur during the close stage and is voltage-independent suggests that its binding site is not within the channel pore. In order to further investigate this, we tested if picrotoxin interacts with the binding of **2c**. Picrotoxin is known to bind into a site within the channel formed by the second transmembrane domains of the five subunits constituting the GABA_A_ receptors [23,24]. We found that neither picrotoxin effect was modified by **2c** nor the inhibitory effect of **2c** was affected by picrotoxin (Figure 9), which would indicate that **2c** does not bind into the picrotoxin binding site.

**Figure 10:**
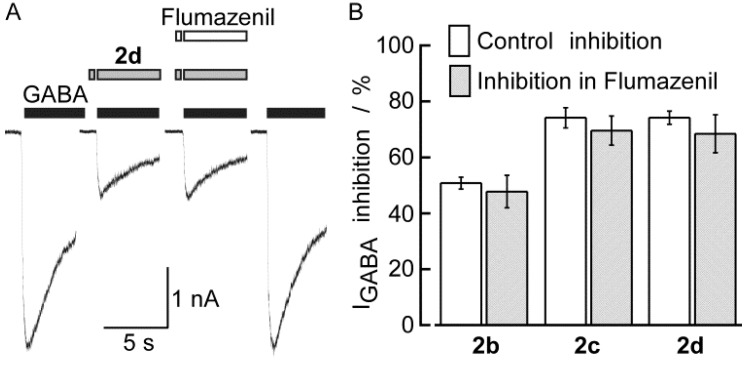
The inhibitory effects of compounds **2** on the GABA_A_ channels were independent of the benzodiazepine binding site. (**A**) First and last traces represent control currents induced by GABA (300 μM). The two middle traces were recorded in **2d** (100 μM) alone or plus flumazenil (10 μM), all traces are from the same neuron. (**B**) Each pair of bars represents the mean inhibition of I_GABA_ induced by DBTDs, before (Control) and in the presence of flumazenil. Lines over the bars indicate the SEM.

The tricyclic sulfonamide **2** is also structurally similar to the 1,4-benzodiazepines, hence, the inhibitory actions of DBTDs may be related to the benzodiazepine modulator sites. The inhibitory effects of **2b**, **2c**, and **2d** remained constant in the absence and presence of flumazenil, a known antagonist of the benzodiazepine site on the GABA_A_ receptors (Figure 10). Inhibition by 100 μM **2b**, **2c**, and **2d** without flumazenil (50.8 ± 2.1%, 74.2 ± 3.6%, 74.2 ± 2.4%, respectively) was not blocked by the co-application of 10 μM flumazenil (47.8 ± 5.8%, 69.6 ± 5.2%, 68.4 ± 6.8%, respectively). The same concentration of flumazenil, applied for 3 min, did not induce changes in the holding current or I_GABA_ in experiments carried out using five different neurons (data not shown). We showed that **2b** did not act through the benzodiazepine binding site, which is in agreement with the lack of binding with receptors to benzodiazepine, previously reported [4].

## 3. Experimental

### 3.1. Chemistry

#### 3.1.1. General

All reagents and solvents were reagent-grade and were used as received from Sigma-Aldrich Co. (St. Louis, MO, USA). Flash chromatography was performed using Merk Kiesegel 60 silica gel (230–400 mesh). Melting points are reported uncorrected. The FTIR spectra were recorded on a Thermo Nicolet Nexus 470 FTIR as thin films on a KBr disk (for solids) or a germanium ATR crystal (for liquids). ^1^H-NMR and ^13^C-NMR spectra were obtained using an Eclipse Jeol (operating at 300 and 75 MHz, respectively) and a Varian-Gemini (operated at 200 MHz and 50 MHz, respectively), and the signals are reported in ppm relative to TMS. All mass spectra (MS) were recorded on a Jeol AX505HA mass spectrometer. Elemental analyses were performed on a CE-440 Exeter Analytical Inc.

#### 3.1.2. General Procedures for Synthesizing *N*-(4-(*R*)-Phenyl)-2-nitrobenzenesulfonamides **5a–f**

Anhydrous pyridine (1.10 mL, 13.6 mmol) and 4-*R*-aniline (1.24 mL, 13.6 mmol) in dry acetone were added, via cannula, to a stirred solution of 2-nitrobenzenesulfonyl chloride (3.01 g, 13.6 mmol) in dry acetone (25 mL) under nitrogen, and the reaction mixture was stirred at room temperature. After 24 h the mixture was neutralized with a saturated sodium bicarbonate solution, and the resulting solid was collected, washed with water and ethanol, and dried under vacuum. The solid was purified by flash chromatography (silica gel, eluting with 90:10 hexane–ethyl acetate), then recrystallized from ethyl acetate–hexane (30:70) to obtain **5a** as a white solid (11.97 mmol, 88%); m.p.: 118–119 °C. The same procedure was used for the synthesis of **5b**–**5f**. Products **5**, **6**, and **4** (except for series **f)** were first reported by Saeed and Rama [25]. However, **5** and **6** compounds were not characterized and **4** was only partially characterized by IR and MS spectroscopies.

*N-(Phenyl)-2-nitrobenzenesulfonamide* (**5a**). ^1^H-NMR (300 MHz, CDCl_3_ + DMSO-*d_6_*): *δ* = 7.08 (m, 1H), 7.21 (m, 4H), 7.63 (ddd, *J_o_* = 7.5 Hz, *J_m_* = 1.6 Hz, 1H), 7.70 (ddd, *J_o_* = 7.5 Hz, *J_m_* = 1.6 Hz, 1H), 7.76 (dd, *J_o_* = 8.0 Hz, *J_m_* = 1.6 Hz, 1H), 7.92 (dd, *J_o_* = 7.9 Hz, *J_m_* = 1.6 Hz, 1H), 9.9 ppm (s, 1H); IR (KBr): ν = 3324, 1380, 1180 cm^−1^; MS (EI, 70 eV): *m/z*: 278 [M]^+^.

*N-(4-Fluorophenyl)-2-nitrobenzenesulfonamide* (**5b**). Yellow crystals. Yield 79%; m.p.: 106 °C; ^1^H-NMR (200 MHz, DMSO-*d_6_*): *δ* = 7.13 (d, *J_o_* = 6.8 Hz, 4H), 7.88 (m, 4H), 10.7 ppm (s, 1H); IR (KBr): ν = 3293, 1360, 1160 cm^−1^; MS (EI, 70 eV): *m/z*: 296 [M]^+^.

*N-(4-Chlorophenyl)-2-nitrobenzenesulfonamide* (**5c**). Yellow crystals. Yield 66%; m.p.: 122 °C; ^1^H-NMR (200 MHz, DMSO-*d_6_*): *δ* = 7.13 (d, *J_o_* = 9.0 Hz, 2H), 7.35 (d, *J_o_* = 9.0 Hz, 2H), 7.89 (m, 4H), 10.9 ppm (s, 1H); IR (KBr): ν = 3309, 1334, 1162 cm^−1^; MS (EI, 70 eV): *m/z*: 312 [M]^+^.

*N-(4-Bromophenyl)-2-nitrobenzenesulfonamide* (**5d**). Colorless crystals. Yield 79%; m.p.: 118 °C; ^1^H-NMR (200 MHz, DMSO-*d_6_*): *δ* = 7.06 (d, *J_o_* = 9.0 Hz, 2H), 7.47 (d, *J_o_* = 8.8 Hz, 2H), 7.91 (m, 4H), 10.9 ppm (s, 1H); IR (KBr): ν = 3297, 1363, 1164 cm^−1^. MS (EI, 70 eV): *m/z*: 358/356 [M]^+^.

*N-(4-Methoxyphenyl)-2-nitrobenzenesulfonamide* (**5e**). Yellow needle crystals. Yield 72%; m.p.: 90 °C; ^1^H-NMR (200 MHz, DMSO-*d_6_*): *δ* = 3.67 (s, 3H), 6.83 (d, *J_o_* = 9.0 Hz, 2H), 7.03 (d, *J_o_* = 9.0 Hz, 2H), 7.87 (m, 4H), 10.4 ppm (s, 1H); IR (KBr): ν = 3259, 1361, 1172 cm^−1^; MS (EI, 70 eV): *m/z*: 308 [M]^+^.

*Ethyl-4-(2-nitrophenylsulfonamido)benzoate* (**5f**). Brown solid. Yield 82%; m.p.: 172 °C; ^1^H-NMR (300 MHz, DMSO-*d_6_*): *δ* = 1.25 (t, *J_o_* = 7.1 Hz, 3H), 4.22 (q, *J_o_* = 7.1 Hz, 2H), 7.13 (BB', *J_o_* = 8.7 Hz, 2H), 7.75 (ddd, *J_o_* = 7.5 Hz, 1H), 7.78 (AA', *J_o_* = 8.7 Hz, 2H), 7.79 (ddd, *J_o_* = 7.5 Hz, 1H), 7.91 (dd, *J_o_* = 6.6 Hz, 1H), 7.98 ppm (dd, *J_o_* = 7.0 Hz, 1H); IR (KBr): ν = 3200, 1690, 1365, 1162 cm^−1^; MS (EI, 70 eV): *m/z*: 350 [M]^+^.

#### 3.1.3. General Procedures for the Synthesis of 2-Amino-*N*-(4-(*R*) phenyl)benzenesulfonamides **6a-f**

*N*-(4-(*R*) phenyl)-2-nitrobenzenesulfonamide **5** (4.3 g, 15.6 mmol) and tin (II) chloride dehydrate (14.82 g, 65.7 mmol) were heated in ethyl acetate under reflux for 4 h. The mixture was stirred, and a saturated sodium bicarbonate solution was added to a pH of 6. The solution was extracted with ethyl acetate. The solvent was removed, and the residue was purified by flash chromatography (eluting with a 90:10 solution of hexane–ethyl acetate).

*2-Amino-N-phenylbenzenesulfonamide* (**6a**). Yellow powder (14.04 mmol, 90%); m.p.: 123–124 °C; ^1^H-NMR (300 MHz, DMSO-*d_6_*): *δ* = 5.99 (s, 2H), 6.54 (ddd, *J_o_* = 7.6 Hz, *J_m_* = 1.2 Hz, 1H), 6.75 (dd, *J_o_* = 8.1 Hz, *J_m_* = 0.9 Hz, 1H), 6.97 (ddd, *J_o_* = 8.2 Hz, 1H), 7.04 (dd, *J_o_* = 8.6 Hz, 2H), 7.2 (m, 3H), 7.49 (dd, *J_o_* = 8.5 Hz, *J_m_* = 1.4 Hz, 1H), 10.2 ppm (s, 1H); IR (KBr): ν = 3457, 3368, 3240, 1360, 1180 cm^−1^; MS (EI, 70 eV): *m/z*: 248 [M]^+^.

*2-Amino-N-(4-fluorophenyl) benzenesulfonamide* (**6b**). Brown liquid. Yield 99%; ^1^H-NMR (200 MHz, DMSO-*d_6_*): *δ* = 5.98 (s, 2H), 6.53 (ddd, *J_o_* = 7.05 Hz, *J_m_* = 1.2 Hz, 1H), 6.74 (dd, *J_o_* = 8.3 Hz, *J_m_* = 1.2 Hz, 1H), 7.05 (d, *J_o_* = 6.4 Hz, 4H), 7.21 (ddd, *J_o_* = 7.0 Hz, *J_m_* = 1.6 Hz, 1H), 7.43 (dd, *J_o_* = 8.2 Hz, *J_m_* = 1.6 Hz, 1H), 10.2 ppm (s, 1H); IR (KBr): ν = 3469, 3382, 3284, 1313, 1147 cm^−1^; MS (EI, 70 eV): *m/z*: 266 [M]^+^.

*2-Amino-N-(4-chlorophenyl) benzenesulfonamide* (**6c**). Brown liquid. Yield 99%; ^1^H-NMR (200 MHz, DMSO-*d_6_* ): *δ* = 6.0 (s, 2H), 6.53 (ddd, *J_o_* = 8.0 Hz, *J_m_* = 1.1, 1H), 6.73 (dd, *J_o_* = 8.3 Hz, *J_m_* = 0.9 Hz, 1H), 7.03 (d, *J_o_* = 8.8 Hz, 2H), 7.2 (ddd, *J_o_* = 8.5 Hz, *J_m_* = 1.6, 1H), 7.26 (d, *J_o_* = 8.8 Hz, 2H), 7.47 (dd, *J_o_* = 8.0 Hz, *J_m_* = 1.6, 1H), 10.4 ppm (s, 1H); IR (KBr): ν = 3467, 3378, 3245, 1313, 1135 cm^−1^; MS (EI, 70 eV): *m/z*: 282 [M]^+^.

*2-Amino-N-(4-bromophenyl) benzenesulfonamide* (**6d**). Brown liquid. Yield 99%; ^1^H-NMR (200 MHz, DMSO-*d_6_*): *δ* = 6.0 (s, 2H), 6.55 (ddd, *J_o_* = 7.8 Hz, 1H), 6.76 (dd, *J_o_* = 8.3 Hz, *J_m_* = 0.9 Hz, 1H), 7.0 (d, *J_o_* = 8.8 Hz, 2H), 7.21 (ddd, *J_o_* = 7.0 Hz, *J_m_* = 1.6 Hz, 1H), 7.39 (d, *J_o_* = 8.8 Hz, 2H), 7.49 (dd, *J_o_* = 7.9 Hz, *J_m_* = 1.5 Hz, 1H), 10.4 ppm (s, 1H); IR (KBr): ν = 3482, 3384, 3268, 1319, 1139 cm^−^^1^; MS (EI, 70 eV): *m/z*: 328/326 [M]^+^.

*2-Amino-N-(4-methoxyphenyl) benzenesulfonamide* (**6e**). Brown liquid. Yield 99%; ^1^H-NMR (200 MHz, DMSO-*d_6_*): *δ* = 3.65 (s, 3H), 5.9 (s, 2H), 6.5 (ddd, *J_o_* = 7.7 Hz, *J_m_* = 1.2 Hz, 1H), 6.72 (dd, *J_o_* = 8.7 Hz, 1H), 6.77 (d, *J_o_* = 9.0 Hz, 2H), 6.95 (d, *J_o_* = 9.0 Hz, 2H), 7.19 (ddd, *J_o_* = 8.2 Hz, *J_m_* = 1.6 Hz, 1H), 7.36 (dd, *J_o_* = 8.1 Hz, *J_m_* = 1.6 Hz, 1H), 9.84 ppm (s, 1H); IR (KBr): ν = 3480, 3380, 3266, 1321, 1147 cm^−1^; MS (EI, 70 eV): *m/z*: 278 [M]^+^.

*Ethyl-4-(2-aminophenylsulfonamido)benzoate* (**6f**). Yellow crystals. Yield 88%; m.p.:163 °C; ^1^H-NMR (200 MHz, DMSO-*d_6_*): *δ* = 1.25 (t, *J_o_* = 6.3 Hz, 3H), 4.22 (q, *J_o_* = 7.0 Hz, 2H), 6.0 (s, 2H), 6.56 (ddd, *J_o_* = 7.6 Hz, 1H), 6.73 (dd, *J_o_* = 7.8 Hz, 1H), 7.14 (BB', *J*_o_ = 8.8 Hz, 2H), 7.22 (ddd, *J_o_* = 7.7 Hz, 1H), 7.57 (dd, *J_o_* = 8.1 Hz, *J_m_* = 1.6 Hz, 1H), 7.79 (AA', *J_o _* = 8.8 Hz, 2H), 10.8 ppm (s, 1H); IR (KBr): ν = 3470, 3380, 3230, 1690, 1322, 1144 cm^−1^; MS (EI, 70 eV): *m/z*: 320 [M]^+^.

#### 3.1.4. General Procedures for Synthesizing 2-Azido-*N*-(4-(*R*)phenyl)benzenesulfonamides **4a-f**

An aqueous solution of sodium nitrite (3.6 g, 51.8 mmol) was added to 2-amino-*N*-(4-(*R*)phenyl)benzenesulfonamide **6** (2.86 g, 11.5 mmol) in trifluoroacetic acid, and the reaction mixture was stirred for 1 h. Sodium azide (1.9 g, 28.8 mmol) was added, and the solution was stirred for an additional 1 h. The mixture was neutralized with saturated sodium bicarbonate solution and extracted with ethyl acetate. The reaction mixture was concentrated and purified by flash chromatography (70:30, hexane–ethyl acetate). The obtained solid was recrystallized in ethyl acetate–hexane (30:70).

*2-Azido-N-phenylbenzenesulfonamide* (**4a**). Brown powder (9.78 mmol, 85%); m.p.:139 °C; ^1^H-NMR (200 MHz, DMSO-*d_6_*): *d* = 6.97 (ddd, *J_o_* = 7.0 Hz, 1H), 7.1 (dd, *J_o_* = 7.4 Hz, 2H), 7.2 (ddd, *J_o_* = 7.4 Hz, 2H), 7.28 (ddd, *J_o_* = 7.6 Hz, 1H), 7.5 (dd, *J_o_* = 8.1 Hz, 1H), 7.64 (ddd, *J_o_* = 7.8 Hz, 1H), 7.86 (dd, *J_o_* = 7.8 Hz, 1H), 10.4 ppm (s, 1H); IR (KBr): ν = 3253, 2133, 1340, 1190 cm^−1^; MS (EI, 70 eV): *m/z*: 274 [M]^+^.

*2-Azido-N-(4-fluorophenyl) benzenesulfonamide* (**4b**). White solid. Yield 83%; m.p.: 129 °C; ^1^H-NMR (200 MHz, DMSO-*d_6_*): *δ* = 7.09 (m, 4H), 7.27 (ddd, *J_o_* = 7.3 Hz, *J_m_* = 1.4 Hz, 1H), 7.52 (dd, *J_o_* = 8.0 Hz, *J_m_* = 1.0 Hz, 1H), 7.62 (ddd, *J_o_* = 8.4 Hz, *J_m_* = 1.6 Hz, 1H), 7.8 (dd, *J_o_* = 7.8 Hz, *J_m_* = 1.6 Hz, 1H), 10.2 ppm (s, 1H); IR (KBr): ν = 3249, 2140, 1334, 1166 cm^−1^; MS (EI, 70 eV): *m/z*: 292 [M]^+^.

*2-Azido-N-(4-chlorophenyl) benzenesulfonamide* (**4c**). White crystals. Yield 80%; m.p.: 134 °C; ^1^H-NMR (200 MHz, DMSO-*d_6_*): *δ* = 7.11 (d, *Jo* = 8.8 Hz, 2H), 7.27 (d, *J_o_* = 8.8 Hz, 2H), 7.31 (ddd, *J_m_* = 1.1 Hz, 1H), 7.51 (dd, *J_o_* = 8.0 Hz, *J_m_* = 1.4 Hz, 1H), 7.66 (ddd, *J_o_* = 7.7 Hz, *J_m_* = 1.6 Hz, 1H), 7.86 (dd, *J_o_* = 7.8 Hz, *J_m_* = 1.4 Hz, 1H), 10.5 ppm (s, 1H); IR (KBr): ν = 3345, 2132, 1338, 1164 cm^−1^; MS (EI, 70 eV): *m/z*: 308 [M]^+^.

*2-Azido-N-(4-bromophenyl) benzenesulfonamide* (**4d**). Yellow powder. Yield 87%; m.p.: 124 °C; ^1^H-NMR (200 MHz, DMSO-*d_6_*): *δ* = 7.05 (d, *J_o_* = 8.8 Hz, 2H), 7.29 (ddd, *J_o_* = 7.0 Hz, *J_m_* = 1.2 Hz, 1H), 7.4 (d, *J_o_* = 8.8 Hz, 2H), 7.52 (dd, *J_o_* = 8.0 Hz, *J_m_* = 1.2 Hz, 1H), 7.67 (ddd, *J_o_* = 6.8 Hz, *J_m_* = 1.4 Hz, 1H), 7.86 (dd, *J_o_* = 7.8 Hz, *J_m_* = 1.6 Hz, 1H), 10.5 ppm (s, 1H); IR (KBr): ν = 3338, 2132, 1338, 1164 cm^−1^; MS (EI, 70 eV): *m/z*: 354/352 [M]^+^.

*2-Azido-N-(4-methoxyphenyl) benzenesulfonamide* (**4e**). Brown crystals. Yield 78%; m.p.: 130 °C; ^1^H-NMR (200 MHz, DMSO-*d_6_*): *δ* = 3.63 (s, 3H), 6.76 (d, *J_o_* = 9.3 Hz, 2H), 7.01 (d, *J_o_* = 8.7 Hz, 2H), 7.23 (ddd, *J_o_* = 7.4, *J_m_* = 1.1 Hz, 1H), 7.51 (dd, *J_o_* = 7.9 Hz, *J_m_* = 1.1 Hz, 1H), 7.62 (ddd, *J_o_* = 7.8 Hz, *J_m_* = 1.6 Hz, 1H), 7.73 (dd, *J_o_* = 7.9 Hz, *J_m_* = 1.2 Hz, 1H), 9.87 ppm (s, 1H); IR (KBr): ν = 3274, 2138, 1336, 1164 cm^−1^; MS (EI, 70 eV): *m/z*: 304 [M]^+^.

*Ethyl-4-(2-azidophenylsulfonamido) benzoate* (**4f**). Brown crystals. Yield 78%; m.p.:182–184 °C; ^1^H-NMR (200 MHz, DMSO-*d_6_*): *δ* = 1.23 (t, *J*_o_ = 7.0 Hz, 3H), 4.20 (q, *J*_o_ = 7.1 Hz, 2H), 7.19 (BB', *J*_o_ = 8.7 Hz, 2H), 7.30 (ddd, *J*_o_ = 7.8 Hz, 1H), 7.48 (dd, *J*_o_ = 8.1 Hz, 1H), 7.65 (ddd, *J*_o_ = 7.8 Hz, 1H), 7.77 (AA', *J*_o_ = 8.7 Hz, 2H), 7.94 (dd, *J*_o_ = 7.8 Hz, 1H), 10.9 ppm (s, 1H); IR (KBr): ν = 3230, 2110, 1690, 1300, 1165 cm^−1^; MS (EI, 70 eV): *m/z*: 346 [M]^+^.

#### 3.1.5. General Procedures for Synthesizing 9-(*R*)-6,11-Dihydrodibenzo[c,f][1,2,5]thiadiazepine-5,5-dioxides **2a–f**

2-Azido-*N*-(4-(*R*)phenyl) benzenesulfonamide **4** (0.2 g, 0.73 mmol) was added to a solution of diphenyl ether (10 mL, 63 mmol) at 208 °C. The solution was stirred for 5 min then cooled to room temperature. The reaction mixture was purified by flash chromatography (70:30, ethyl acetate–hexane). The resulting residue was recrystallized in ethyl acetate–hexane (30:70). Compounds **2a** and **2d** were previously reported by Altamura *et al.* [8], and spectroscopic data are in agreement with those reported here.

*6,11-Dihydrodibenzo[c,f][1,2,5]thiadiazepine-5,5-dioxide* (**2a**). Brown crystals (0.50 mmol, 69%); m.p.: 198 °C; ^1^H-NMR (300 MHz, DMSO-*d_6_*): *δ* = 6.82 (ddd, *J_o_* = 7.8 Hz, *J_m_* = 0.9 Hz, 1H), 6.88 (dd, *J_o_* = 7.4 Hz, *J_m_* = 1.5 Hz, 1H), 7.03 (ddd, *J_o_* = 7.7 Hz, *J_m_* = 1.5 Hz, 1H), 7.07 (dd, *J_o_* = 8.1 Hz, *J_m_* = 1.5 Hz, 1H), 7.14 (ddd, *J_o_* = 7.5 Hz, *J_m_* = 1.5 Hz, 1H), 7.18 (dd, *J_o_* = 8.2 Hz, *J_m_* = 1.0 Hz, 1H), 7.37 (ddd, *J_o_* = 7.7 Hz, *J_m_* = 1.6 Hz, 1H), 7.61 (dd, *J_o_* = 8.0 Hz, *J_m_* = 1.6 Hz, 1H), 8.98 (s, 1H), 9.80 ppm (s, 1H); ^13^C-NMR (75 MHz, DMSO-*d_6_*): *δ* = 117.5, 119.5, 119.6, 121.1, 125.2, 125.9, 127.3, 128.4, 128.8, 132.9, 139.5, 139.9 ppm; IR (KBr): ν = 3380, 3301, 1313, 1160 cm^−1^; MS (EI, 70 eV): *m/z*: 246 [M]^+^; Anal. calculated for C_12_H_10_N_2_O_2_S: C 58.52%, H 4.09%, N 11.37%, found: C 58.11%, H 4.11%, N 11.13%.

*9-Fluoro-6,11-dihydrodibenzo[c,f][1,2,5]thiadiazepine-5,5-dioxide* (**2b**). Colorless needle crystals. Yield 70%; m.p.: 202 °C; ^1^H-NMR (300 MHz, DMSO-*d_6_*): *δ* = 6.69 (ddd, *J_o_* = 8.2 Hz, *J_mH-F_* = 3.0 Hz, 1H), 6.86 (dd, *J_o_* = 7.2 Hz, 1H), 6.89 (d, *J_m_* = 2.7 Hz, 1H), 7.05 (dd, *J_o_* = 7.5 Hz, 1H), 7.16 (dd, *J_o_* = 8.1 Hz, *J_m_* = 0.6 Hz, 1H), 7.41 (ddd, *J_o_* = 7.6 Hz, *J_m_* = 1.5 Hz, 1H), 7.63 (dd, *J_o_* = 8.0 Hz, *J_m_* = 1.8 Hz, 1H), 9.14 (s, 1H), 9.78 ppm (s, 1H); ^13^C-NMR (75 MHz, DMSO-*d_6_*): *δ* = 105.6, 107.6, 119.0, 121.4, 126.1, 129.3, 130.4, 133.1, 139.2, 141.3, 159.2, 162.5 ppm; IR (KBr): ν = 3365, 3226, 1295, 1159 cm^−1^; MS (EI, 70 eV): *m/z*: 264 [M]^+^; Anal. calculated for C_12_H_9_N_2_O_2_SF: C 54.54%, H 3.43%, N 10.60%, found: C 54.05%, H 3.32%, N 10.34%.

*9-Chloro-6,11-dihydrodibenzo[c,f][1,2,5]thiadiazepine-5,5-dioxide* (**2c**). White powder. Yield 79%; m.p.: 248 °C; ^1^H-NMR (300 MHz, DMSO-*d_6_*): *δ* = 6.87 (ddd, *J_o_* = 7.8 Hz, *J_m_* = 1.2 Hz, 1H), 6.88 (dd, *J_o_* = 8.4 Hz, *J_m_* = 2.1 Hz, 1H), 7.01 (d, *J_o_* = 8.4 Hz, 1H), 7.13 (d, *J_m_* = 2.1 Hz, 1H), 7.15 (dd, *J_o_* = 8.1 Hz, *J_m_* = 0.6 Hz, 1H), 7.41 (ddd, *J_o_* = 7.7 Hz, *J_m_* = 1.5 Hz, 1H), 7.62 (dd, *J_o_* = 8.0 Hz, *J_m_* = 1.6 Hz, 1H), 9.11 (s, 1H), 9.95 ppm (s, 1H); ^13^C-NMR (75 MHz, DMSO-D_6_): *δ* = 118.3, 118.7, 119.7, 120.4, 124.2, 125.8, 129.2, 129.5, 131.1, 133.3, 139.2, 140.5 ppm; IR (KBr): ν = 3369, 3269, 1304, 1156cm^−1^; MS (EI, 70 eV): *m/z*: 280 [M]^+^; Anal. calculated for C_12_H_9_N_2_O_2_SCl: C 51.34%, H 3.23%, N 9.98%, found: C 51.03%, H 3.22%, N 9.81%.

*9-Bromo-6,11-dihydrodibenzo[c,f][1,2,5]thiadiazepine-5,5-dioxide* (**2d**). Brown powder. Yield 85%; m.p.: 250 °C; ^1^H-NMR (300 MHz, DMSO-*d_6_*): *δ* = 6.87 (ddd, *J_o_* = 7.4 Hz, 1H), 6.94 (d, *J_o_* = 8.4 Hz, 1H), 7.01 (dd, *J_o_* = 8.2 Hz, *J_m_* = 2.0 Hz, 1H), 7.15 (d, *J_o_* = 8.4 Hz, 1H), 7.28 (d, *J_m_* = 1.8 Hz, 1H), 7.41 (ddd, *J_o_* = 7.8 Hz, 1H), 7.62 (dd, *J_o_* = 7.8 Hz, *J_m_* = 1.2 Hz, 1H), 9.10 (s, 1H), 9.96 ppm (s, 1H); ^13^C-NMR (75 MHz, DMSO-*d_6_*): *δ* = 118.3, 119.2, 119.7, 121.6, 123.3, 124.6, 125.8, 129.2, 129.7, 133.3, 139.2, 140.7 ppm; IR (KBr): ν = 3371, 3268, 1308, 1160 cm^−1^; MS (EI, 70 eV): *m/z*: 326/324 [M]^+^; Anal. calculated for C_12_H_9_N_2_O_2_SBr: C 44.32%, H 2.79%, N 8.61%, found: C 44.09%, H 2.81%, N 8.74%.

*9-Methoxy-6,11-dihydrodibenzo[c,f][1,2,5]thiadiazepine-5,5-dioxide* (**2e**). Yellow crystals. Yield 67%; m.p.: 171 °C; ^1^H-NMR (300 MHz, DMSO-*d_6_*): *δ* = 3.73 (s, 3H), 6.49 (dd, *J_o_* = 8.7 Hz, *J_m_* = 2.7 Hz, 1H), 6.66 (d, *J_m_* = 2.7 Hz, 1H), 6.83 (ddd, *J_o_* = 7.5 Hz, *J_m_* = 0.9 Hz, 1H), 6.96 (d, *J_o_* = 8.7 Hz, 1H), 7.17 (d, *J_o_* = 7.8 Hz, 1H), 7.37 (ddd, *J_o_* = 7.7 Hz, *J_m_* = 1.5 Hz, 1H,), 7.62 (dd, *J_o_* = 7.8 Hz, *J_m_* = 1.5 Hz, 1H), 8.99 (s, 1H), 9.51 ppm (s, 1H); ^13^C-NMR (75 MHz, DMSO-D_6_): *δ* = 55.2, 104.2, 107.4, 117.7, 118.1, 119.5, 126.3, 129.0, 130.4, 132.8, 139.7, 141.2, 158.6 ppm; IR (KBr): ν = 3374, 3228, 1322, 1149 cm^−1^; MS (EI, 70 eV): *m/z*: 276 [M]^+^; Anal. calculated for C_13_H_12_N_2_O_3_S: C 56.51%, H 4.38%, N 10.14%, found: C 56.56%, H 4.40%, N 9.89%.

*Ethyl 6,11-dihydrodibenzo[c,f][1,2,5]thiadiazepine-9-carboxylate-5,5-dioxide* (**2f**). Brown crystals. Yield 12%; m.p.: 227 °C; ^1^H-NMR (300 MHz, DMSO-*d_6_*): *δ* = 1.31 (t, *J*_o_ = 7.0 Hz, 3H), 4.30 (q, *J*_o_ = 7.2 Hz, 2H), 6.85 (ddd, *J*_o_ = 7.5 Hz, 1H), 7.07 (d, *J*_o_ = 8.1 Hz, 1H), 7.19 (d, *J*_o_ = 7.8 Hz, 1H), 7.40 (dd, *J*_o_ = 8.1 Hz, 1H), 7.40 (ddd, *J*_o_ = 7.6 Hz, 1H), 7.61 (dd, *J*_o_ = 7.8 Hz, *J_m_* = 1.5 Hz, 1H), 7.73 (d, *J_m_* = 1.8 Hz, 1H), 9.20 (s, 1H), 10.3 ppm (s, 1H); ^13^C-NMR (75 MHz, DMSO-*d_6_*): *δ* = 14.2, 60.7, 118.1, 119.6, 120.4, 121.3, 125.3, 126.8, 128.2, 128.9, 129.6, 133.4, 138.3, 139.6, 165.1 ppm; IR (KBr): ν = 3360, 3234, 1700, 1328, 1170 cm^−1^; MS (EI, 70 eV): *m/z*: 318 [M]^+^; Anal. calculated for C_15_H_14_N_2_O_4_S: C 56.59%, H 4.43%, N 8.80%, found: C 56.67%, H 4.44%, N 8.64%.

#### 3.1.6. Procedure for Synthesizing 6,11-Dihydrodibenzo[c,f][1,2,5]thiadiazepine-9-carboxylic acid 5,5-dioxide (**2g**)

A solution of potassium hydroxide 10% (w/v) was added to ethyl 6,11-dihydrodibenzo[*c*,*f*][1,2,5]thiadiazepine-9-carboxylate-5,5-dioxide (**2f**, 0.5 g, 1.57 mmol). The reaction mixture was heated under reflux and stirred for 60 min, after which a chloride acid solution was added to a pH of 6. The resulting solid was collected, washed with water, and dried under vacuum. The resulting yellow solid residue was obtained in a quantitative yield; m.p.: 350 °C; ^1^H-NMR (300 MHz, DMSO-*d_6_*): *δ* = 6.84 (ddd, *J_o_* = 7.6 Hz, *J_m_* = 1.0 Hz, 1H), 7.05 (d, *J_o_* = 8.1 Hz, 1H), 7.18 (d, *J_o_* = 8.1 Hz, 1H), 7.39 (dd, *J_o_* = 8.1 Hz, *J_m_* = 1.8 Hz, 1H), 7.39 (ddd, *J_o_* = 7.6 Hz, *J_m_* = 1.8 Hz, 1H), 7.61 (dd, *J_o_* = 8.0 Hz, *J_m_* = 1.6 Hz, 1H), 7.72 (d, *J_m_* = 1.8 Hz, 1H), 9.15 (s, 1H), 10.7 ppm (s, 2H); ^13^C-NMR (75 MHz, DMSO-*d_6_*): *δ* = 118.0, 119.6, 120.7, 121.6, 125.4, 126.8, 128.9, 129.3, 129.4, 133.4, 138.3, 139.7, 166.7 ppm; IR (KBr): ν = 3360, 3234, 1700, 1328 cm^−1^; MS (EI, 70 eV): *m/z*: 290 [M]^+^; Anal. calculated for C_13_H_10_N_2_O_4_S: C 53.79%, H 3.47%, N 9.65%, found: C 53.73%, H 3.47%, N 9.28%.

### 3.2. Biological Methods

#### 3.2.1. Primary Cultures of the Myenteric Neurons

Guinea pigs (100–200 g; either male or female) were sacrificed by cervical dislocation and carotid exsanguination. These methods have been approved by the Animal Care Committee of the IPICYT and are in agreement with the published Guiding Principles in the Care and Use of Animals, approved by the American Physiological Society. A segment of ~10 cm of the jejunum was removed and placed in a modified Krebs solution (in mM: NaCl, 126; NaH_2_PO_4_, 1.2; MgCl_2_, 1.2; CaCl_2_, 2.5; KCl, 5; NaH_2_CO_3_, 25; glucose, 11. The sample was gassed under 95% O_2_ and 5% CO_2_) and opened longitudinally. A dissecting microscope was used to dissect the mucosa and submucosa layers prior to removing most of the circular muscle layer, leaving the myenteric plexus embedded in a longitudinal layer.

The cell isolation procedure has been described elsewhere [26]. The myenteric preparation was dissociated by sequential treatment with two enzymatic solutions: the first solution contained papain (0.01 mL·mL^−1^ activated with 0.4 mg·mL^−1^L-cysteine), and the second solution contained collagenase (1 mg·mL^−1^) and dispase (4 mg·mL^−1^). The enzymes were removed by washing the neurons with L15 medium, and the neurons were placed on round coverslips coated with sterile rat-tail collagen. The culture medium was varied from minimal medium to essential medium 97.5% containing 2.5% guinea pig serum, 2 mM L-glutamine, 10 U·mL^−1^ penicillin, 10 μg·mL^−1^ streptomycin, and 15 mM glucose.

#### 3.2.2. Whole-Cell Recordings of the Membrane Currents Induced by GABA

To reduce the effects of the membrane currents other than those mediated by the activation of LGIC, experiments were conducted in the presence of Cs^+^ (a potassium channel blocker). This was important because GABA modulates the membrane ion channels of the central neurons (enteric neurons) via G-protein linked receptors [27,28,29]. Membrane currents induced by GABA were recorded using a Gene Clamp 500B amplifier (Molecular Devices, CA, USA). The holding potential was −60 mV (unless otherwise stated), and the short-term (4–50 h) primary cultures of the myenteric neurons were used to prevent space-clamp problems due to neurite growth. Glass pipettes with a resistance of 2–5 MΩ were prepared as described previously [25]. This low resistance and slight suction inside the pipette during the recordings maintained a low series resistance (around 6 MΩ).

All experiments were conducted using standard solutions with the following compositions (in mM); inside the pipette: CsCl, 160; EGTA, 10; HEPES, 5; NaCl, 10; ATPMg, 3 and GTP, 0.1; external solution: NaCl, 160; CaCl_2_, 2; glucose, 11; HEPES, 5 and CsCl, 3. The pH of all solutions was adjusted to 7.3–7.4 using either CsOH (pipette solution) or NaOH (external solution). The seal resistance in the whole-cell mode ranged from 1 to 10 GΩ. The whole-cell current data were recorded on a PC using the AxoScope software (Axon Instruments, Inc.) and were analyzed using the AXOGRAPH software (Molecular Devices, CA, USA). The recording chamber was superfused with an external solution at ~2 mL·min^−1^. The solution around the neuron was quickly exchanged during recordings using an eight-tube device. Each tube was connected to a syringe (10 mL) containing either the control or the experimental solution. A control tube was positioned ~300 μm in front of the recorded neuron, and substances were applied externally by abruptly interchanging the tube for another tube containing the control solution plus the drug(s). Desensitization of the GABA_A_ receptors was prevented by applying GABA at intervals of at least 5 min, in between cells were continuously superfused with extracellular solution. Experimental substances were removed by returning to the control solution. External solutions were applied by gravity, and the height of the syringes was continuously adjusted to minimize changes in the flow rate. The experiments were performed at room temperature (24 ± 1 °C).

#### 3.2.3. Solutions and Reagents

L15 medium, minimum essential medium, Hanks solution, penicillin-streptomycin, and L-glutamine were purchased from GIBCO (Life Technologies Corp., Carlsbad, CA, USA). Collagenase and papain were purchased from Worthington (Worthington Biochemical Corp., Lakewood, NJ, USA), and dispase was purchased from Roche (Indianapolis, IN, USA). Cesium chloride, sodium chloride, ethylene glycol-bis(2-aminoethylether)-*N,N,N',N'*-tetra-acetic acid (EGTA), HEPES, adenosine-5*'*-triphosphate magnesium salt (ATP magnesium salt), guanosine-5*'*-triphosphate sodium salt (GTP sodium salt), cesium hydroxide, flumazenil, GABA, picrotoxin, and dimethyl sulfoxide were purchased from Sigma-Aldrich (St. Louis, MO, USA). Pentobarbital-Na was purchased from Lab Ttokkyo, S.A. (México, D.F., Mexico). Stock solutions (0.01–1 M) were prepared using de-ionized distilled water and were stored frozen, except for picrotoxin and the DBTD stock solutions, which were prepared in ethanol (50% v/v) and DMSO, respectively. The desired final drug concentration was obtained by diluting the stock solutions in an external solution prior to application.

#### 3.2.4. Data Analysis

The concentration–response data were fit to a logistic model:

I = I_max_/[1 + (EC_50_/[A])^nH^] (1)

where [A] is the agonist concentration, I is the current, and I_max_ is the maximum current. EC_50_ is the concentration of drug that elicits a half-maximum response, and nH is the Hill coefficient. Experimental data were reported as ± SEM, and n represents the number of cells used. The unpaired Student’s t-test was applied to data obtained from two different groups of cells. One-way ANOVA and the Bonferroni tests were used to compare multiple means. The two-tailed *P* values of 0.05 or less were considered to be statistically significant.

#### 3.2.5. Theoretical Calculations

Quantum chemical calculations of the DBTDs structures **2a**–**g** in the gas phase were performed using GAUSSIAN 03 [30] in conjunction with density functional theory (DFT) calculations. Geometry optimization of the DBTDs was followed by frequency calculations performed at the B3LYP/6-311 ++G(d,p) level. Table 1 reports the cLog *P*, generated using HyperChem, of each optimized structure obtained from Gaussian V03.

## 4. Conclusions

We described the preparation of novel DBTDs via a nitrene radical that inserted into the C on the aromatic ring. Seven derivatives **2a**–**g** were generated and their effects on GABA_A_ neuronal receptors were tested. It was shown, for the first time, that DBTDs inhibited I_GABA_ in a time- and concentration-dependent manner.

The DBTDs displayed an inhibitory effect on the GABA_A_ channel of myenteric neurons, and this antagonism was non-competitive indicating that it does not bind to the GABA receptor and their effect is likely allosteric. Inhibition was mediated by an extracellular binding site that was most likely not in the mouth of the channel and therefore, it is unlikely that this effect is mediated by channel blockage. Their effect was also independent of the benzodiazepine binding site. The DBTDs described here could be used as a model to explore new GABA_A_ receptor inhibitors with a potential to be used as antidotes for substances known to positively modulate GABA_A_ channel activity or as a new drugs to induce experimental epilepsy. These compounds appear to bind on a different site than picrotoxin; therefore, they could be used alone or in combination with picrotoxin. Future experiments will be aimed to molecularly identify DBTDs binding sites on GABA_A_ receptors.
